# The Role of the IL-33/ST2 Axis in CpG-Induced Macrophage Activation Syndrome

**DOI:** 10.1155/2023/2689360

**Published:** 2023-10-05

**Authors:** Yuanji Dong, Rongfen Gao, Kailin He, Jixin Zhong, Lingli Dong

**Affiliations:** ^1^Department of Rheumatology and Immunology, Tongji Hospital, Tongji Medical College, Huazhong University of Science and Technology, Wuhan, Hubei, China; ^2^Department of Rheumatology, The Second Affiliated Hospital of Zhejiang University School of Medicine, Hangzhou, Zhejiang, China

## Abstract

**Background:**

Macrophage activation syndrome (MAS) is a fatal inflammatory condition, which is often associated with the elevation of multiple proinflammatory cytokines and multiple organ dysfunction. Previous studies have shown that ST2 contributes to T cell overactivation and plays a detrimental role in mouse models of primary hemophagocytic lymphohistiocytosis. The purpose of this study was to investigate the role of the IL-33/ST2 axis in a mouse model of MAS induced by repeated injections of cytosine–phosphate–guanine (CpG).

**Methods:**

Serum cytokines were determined using the cytometric bead array by flow cytometry. IL-33 and ST2 were detected by immunohistochemistry and real-time quantitative PCR in the liver and spleen of mice. CD3 and F4/80 in the liver were detected by immunohistochemistry. Inflammatory macrophages and effector memory T lymphocytes were detected by flow cytometry.

**Result:**

The CpG-induced MAS model was successfully induced after repeated CpG injections, presenting with hypercytokinemia and hepatosplenomegaly. The numbers of IL-33 positive cells in the liver and spleen decreased significantly, while the expression of ST2 in the liver tended to increase in the mice with MAS. *IL-33* and *St2* knockout mice showed similar levels of hepatosplenomegaly, peripheral blood count, and cytokine storm when compared with wild-type (WT) mice after induction of MAS. There were also no significant differences in liver pathology (including inflammatory cell infiltration of CD3 and F4/80) and levels of splenic inflammatory macrophages and effector memory T cells between the WT and knockout mice.

**Conclusion:**

These results suggested that IL-33 decreased in the liver and spleen tissues of MAS mice. Further results suggest that *IL-33* and *St2* knockout mice have no treatment potential in CpG-induced MAS. Thus, the IL-33/ST2 axis has little effect on the prognosis of CpG-induced MAS.

## 1. Introduction

Hemophagocytic lymphohistiocytosis (HLH) can be divided into primary and secondary HLH [1]. Primary HLH is caused by defects in immune-related genes, such as PRF1, Lyst, RAB27A, UNC13D, STXBP2, and STX11. These defective genes lead to the disruption of perforin-mediated lysis, which prevents the effective clearance of infected cells and hyperactivated antigen-presenting cells (APC). As a result, the constant interaction between lymphocytes and APC leads to cytokine storms and organ damage [2]. Macrophage activation syndrome (MAS) is a life-threatening complication of systemic inflammatory disease. It commonly occurs in patients with systemic juvenile idiopathic arthritis and adult still disease [3, 4], as well as other systemic diseases, including but not limited to systemic lupus erythematosus [5], Kawasaki disease [6], malignant tumors, infections, and primary immunodeficiency [7, 8]. Patients diagnosed with MAS are often accompanied by hypercytokinemia, immune system dysfunction, and multiorgan disorders, with high mortality and limited treatment options [9]. A variety of cytokines are involved in the progression of MAS, especially TNF, interferon, IL-6, IL-18, and IL-1. Blockade of these cytokines has shown protective effects on MAS in several studies. Among them, TNF-*α* inhibitor (etanercept) [10], IFN-*γ* monoclonal antibody (Emapalumab) [11], and IL-6 monoclonal antibody (Tocilizumab) [12], recombinant IL-1 receptor antagonists (Anakinra) [13], and pan-Jaki (Ruxolitinib) [14–17] have shown the therapeutic effects in clinical practice. However, due to the complexity of the immune microenvironment, there are still a significant number of patients who are resistant to these treatments [18].

IL-33 is a member of the IL-1 family and is mainly expressed in epithelial cells, endothelial cells, and fibroblasts. IL-33 is also believed to be expressed by macrophages and dendritic cells. Under conditions of cell damage or infection, IL-33 can be released into the extracellular microenvironment and functions as a proinflammatory factor. Upon binding to its receptor ST2, also known as interleukin 1 receptor-like 1, IL-33 has been demonstrated to play an important role in antiparasite immunity [19], allergic reaction [20], asthma [21], organ transplantation [22], tissue fibrosis [23], and autoimmune diseases [24]. Previous studies have shown that IL-33 and ST2 are involved in the development of primary HLH [25–27]. Infection with lymphocytic choroid from meningitis virus (LCMV) in *Prf1*^*−/−*^ mice led to the overactivation of macrophages and T lymphocytes. This process releases large amounts of inflammatory cytokines. Tissue damage and cell death also released IL-33, which could directly or indirectly act on LCMV-specific T lymphocytes to further promote the production of IFN-*γ*, while in turn, further promoting tissue damage and releasing more IL-33, forming positive feedback. Therefore, in the mouse model of primary HLH, IL-33/ST2 is thought to aggravate tissue damage and can be used as a therapeutic target [25]. However, some MAS patients do not have genetic defects but are often induced by infection, and also show hypercytokinemia. For these patients, it remains unclear whether the IL-33/ST2 axis is involved in the development of the disease or whether it can be used as a therapeutic target.

To address this question, we used cytosine–phosphate–guanine (CpG) 1826 as an inducer to mimic the symptoms of MAS in a mouse model and quantify its significance through expression changes and knockout verification.

## 2. Methods

### 2.1. MAS Mice Model Induction

Eight-week-old mice were randomly assigned into control and CpG groups. The mice in the control group were given phosphate-buffered saline (PBS) through intraperitoneal injection, while the mice in the CpG group were given 50 *μ*g CpG 1826 every other day five times in total. The whole blood was harvested from anesthetized mice via the vena cava. The liver (spleen) index was measured by the liver (spleen) weight/body weight × 100. This study was approved by the Ethics Committee of Tongji Hospital.

### 2.2. Cytokines Measurement

Plasma concentrations of cytokines (IL-6, IL-10, MCP1, TNF-*α*, IFN-*γ*, and IL12p70) were detected using the Mouse Inflammation cytometric bead array (CBA) kit (BD) according to the manual. The concentrations were evaluated by flow cytometry. IL-18 was evaluated by a commercial ELISA kit according to the manufacturer's instructions (CUSABIO).

### 2.3. Immunohistochemistry

Formalin-fixed, paraffin-embedded sections were deparaffinized, rehydrated, and subjected to citric acid (pH = 6) or EDTA (pH = 9) microwave antigen retrieval. Slides were blocked with 3% H_2_O_2_ for 60 min to remove endogenous catalase, washed three times with TBS with 0.1% Tween 20 for 5 min, blocked with a 5% BSA for 1 hr at room temperature, and then incubated overnight at 4°C with primary antibodies as listed: 1 mg/ml IL-33 (R&D), ST2 (ABclonal), CD3 (ABclonal), or F4/80 (Proteintech). Slides were then washed three times in TBS with 0.1% Tween 20 for 5 min, incubated with horseradish peroxidase (HRP)-linked donkey antigoat or HRP-linked donkey antirabbit IgG (Proteintech) secondary antibodies for 1 hr at room temperature, washed with TBS with 0.1% Tween 20 for 5 min three times, incubated with diaminobenzidine for 1 min, and then stained with hematoxylin for 30 s. All images were acquired using an Olympus microscope.

### 2.4. Real-Time Quantitative PCR

Total RNA was extracted from the liver or spleen by Trizol reagent (Thermo Fisher), and cDNA was synthesized using a reverse transcription kit (Thermo Fisher). The expressions of *St2*, *Il-33*, and *Actb* were determined by SYBR Green quantitative real-time PCR (Toyobo, Osaka, Japan). The relative gene expression was calculated by using the 2^−*ΔΔ*Ct^ method. The primer sequences were listed as follows: *St2* (forward, 5′-GAATGGGACTTTGGGCTTTG-3′, reverse, 5′-CCATTCCACAGGATAAGTCGAG-3′), *IL-33* (forward 5′-TTCCAACTCCAAGATTTCCCC-3′, reverse, 5′-CAGAACGGAGTCTCATGCAG-3′). *Actb* (forward, 5′-GGTCAGAAGGACTCCTATGTGG-3′, reverse, 5′-TGTCGTCCCAGTTGGTAACA-3′).

### 2.5. Liver Enzyme and Peripheral Blood Tests

The whole blood of MAS and control mice was collected and anticoagulated with sodium citrate. Peripheral blood was sent for examination within 4 hr to obtain red blood cell (RBC) counts, hemoglobin, platelets, and white blood cell counts in peripheral blood. Then the serum was centrifuged at 3,000 rpm for 10 min to detect alanine aminotransferase (ALT) and aspartate aminotransferase (AST) levels.

### 2.6. Histopathological Examination

Paraffin sections of mice organs (liver or spleen) were dewaxed with xylene and rinsed with ethanol. Then the sections were rinsed with deionized water, followed by counterstaining with hematoxylin. Next, the sections were differentiated with 1% hydrochloric acid alcohol for 20 s, treated with 1% ammonia for 30 s, and rinsed with deionized water. Then the tissue sections were stained with eosin solution for 3 min, washed with water, 70% alcohol for 1 min, 90% alcohol, and anhydrous ethanol for 1 min, dried, sealed, and collected pictures under a microscope. For the H&E stained sections of mice liver, the aggregation of eight or more inflammatory cells was called one focus, and the corresponding foci were counted in three random fields under a low-power microscope (×40).

### 2.7. Flow Cytometry

Mouse spleens were homogenized, and single-cell suspensions were prepared and counted after lysis with RBC lysis buffer (BioLegend). For macrophage staining, 1 × 10^6^ cells were incubated with FITC-F4/80 (Biolegend) and APC-Ly6c (Biolegend) antibodies at room temperature for 30 min. The cells were rinsed with 1 ml PBS and resuspended with 200 *μ*l PBS for detection. For the detection of Tcm (central memory T cell) and Tem (effector memory T cell), 1 × 10^6^ cells were incubated with FITC-CD62L (Biolegend), PE-CD44 (Biolegend), Percp-cy5.5-CD4 (Biolegend), and APC-CD8 (Biolegend) antibodies for 30 min. The cells were then subjected to 1 ml PBS washing, followed by suspending with 200 *μ*l PBS for cytometry detection. For intracellular staining, 2 × 10^6^ cells were stimulated with PMA plus ionomycin (Invitrogen) for 5 hr. After incubation with FITC-CD4 (Biolegend) and PE-CD8 (Biolegend) antibodies at room temperature for 30 min, the cells were then incubated with Fix/Perm according to kit instructions and APC-IFN-*γ* (Biolegend) antibodies for 30 min. Then the cells were rinsed with 1 ml PBS and resuspended with 200 *μ*l PBS for detection.

### 2.8. Statistic Analysis

The normality of the distribution of each variable was assessed using the Shapiro–Wilk normality test. Data that presented a normal distribution were analyzed through univariate analysis of variance (ANOVA-one way), followed by the Turkey post-test to determine the differences among the four groups. Data were presented as mean ± standard error. Differences were considered significant when *P* < 0.05. All tests were performed in GraphPad Prism version 7.0 (GraphPad Software, La Jolla).

## 3. Result

### 3.1. Repeated Injection of CpG Successfully Induced MAS

**Figure 1:**
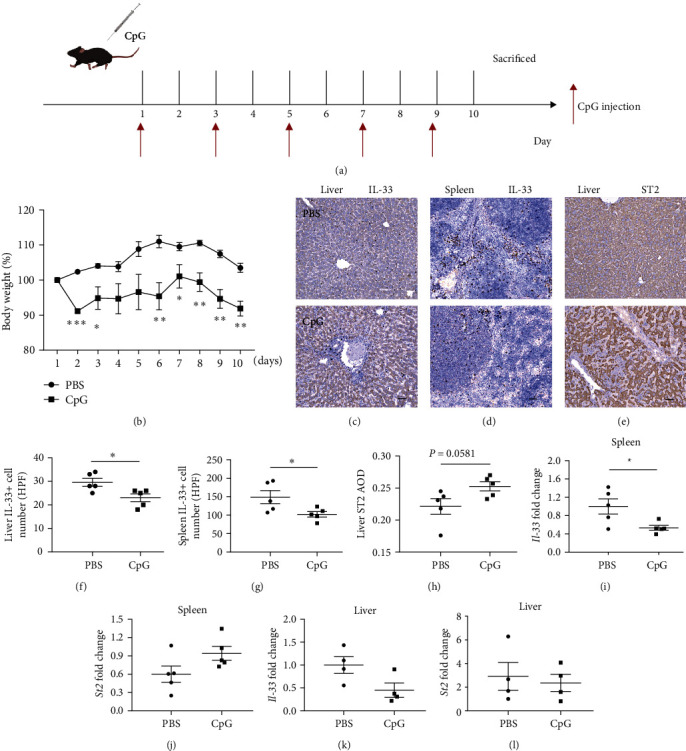
IL-33 and ST2 levels in the liver and spleen of mice 10 days after the model. (a) Flow chart of MAS model induced by repeated CpG in mice. (b) Body weight changes in control mice and mice repeatedly stimulated with CpG. (c) The expression of IL-33 in the liver of control mice and mice was repeatedly stimulated with CpG (bar = 50 *μ*m). (d) The expression of IL-33 in the control mice and mice spleen was repeatedly stimulated with CpG (bar = 50 *μ*m). (e) The expression of ST2 in the liver of control mice and mice was repeatedly stimulated with CpG (bar = 50 *μ*m). (f and g) IL-33 positive cells at high magnification in the liver and spleen of control mice and mice repeatedly stimulated with CpG. (h) The mean optical density of ST2 in the liver of control mice and mice repeatedly stimulated with CpG. (i and j) IL-33 and ST2 transcription levels in control mice and mice spleen were repeatedly stimulated with CpG. (k and l) IL-33 and ST2 transcription levels in the liver of control mice and the CpG-stimulated mice.  ^*∗*^*P* < 0.05,  ^*∗∗*^*P* < 0.01,  ^*∗∗∗*^*P* < 0.001.

Repeated toll-like receptor 9 activation with CpG is a frequently used strategy to induce the MAS mice model. In the present study, the wild-type (WT) mice were intraperitoneally injected with CpG every other day, and samples were collected after five injections in 10 days (Figure 1(a)). The body weight of MAS mice decreased significantly (Figure 1(b)). Spleen and liver were enlarged in model mice, and the liver and spleen indices were also considerably increased (Figure S1(a)–S1(c)). At the same time, the serum levels of IL-10, MCP-1, and TNF-*α* were significantly increased and the levels of IL-6 and IFN-*γ* also tended to increase in MAS mice, but there was no difference in IL-12 between the two groups (Figure S1(d)–S1(i)). These results suggest that repeated CpG injection can induce hypercytokinemia, hepatosplenomegaly, and weight loss, which partially mimic some symptoms of patients with MAS.

### 3.2. Expression Changes of IL-33 and ST2 in MAS Model

Considering the potential roles of the IL-33/ST2 axis in the pathogenesis of MAS, the expression profiles of IL-33 and ST2 in the affected tissues of MAS mice were determined. The immunohistochemical analysis of IL-33 in liver and spleen tissue sections and analysis of the number of positive cells showed that the numbers of IL-33 positive cells in the spleen and liver were significantly reduced after CpG induction (Figures 1(c), 1(d), 1(f), and 1(g)). Furthermore, the average optical density of ST2 in liver tissue sections was increased after CpG administration (Figures 1(e) and 1(h)). Furthermore, real-time quantitative PCR experiments showed that the mRNA expression of *IL-33* in the spleen significantly decreased and the expression of *IL-33* in the liver tended to decrease after CpG induction, while the expression of *St2* showed no difference in liver and spleen (Figure 1(i)–1(l)). These results suggest that the IL-33/ST2 axis may be involved in MAS, however, the underlying mechanisms remain to be elucidated.

### 3.3. IL-33^−/−^ and St2^−/−^ Mice Still Responded Positively to CpG-Induced Immune Activation

**Figure 2:**
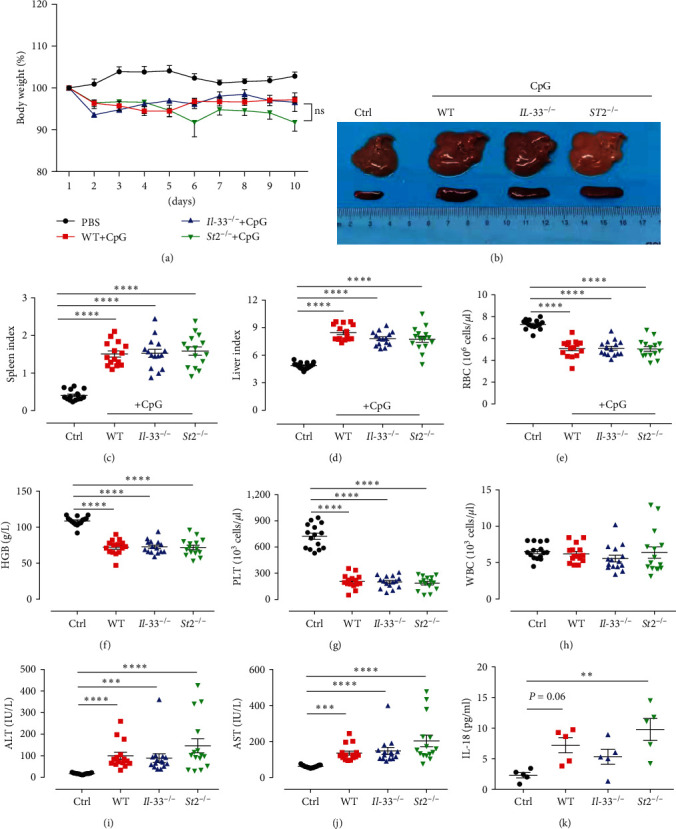
Hepatosplenomegaly and the peripheral blood cell count levels in the four groups of mice 10 days after the model. (a) Weight changes in control mice and CpG repeatedly stimulated mice. (b) Image of liver and spleen in the four groups of mice. (c and d) Spleen index and liver index in four groups of mice. (e–h) Levels of red blood cells, hemoglobin, platelets, and white blood cells in all four groups of mice. (i and j) ALT and AST levels in four groups of mice. (k) Levels of IL-18 in all four groups of mice.  ^*∗∗*^*P* < 0.01,  ^*∗∗∗*^*P* < 0.001,  ^*∗∗∗∗*^*P* < 0.0001.

**Figure 3:**
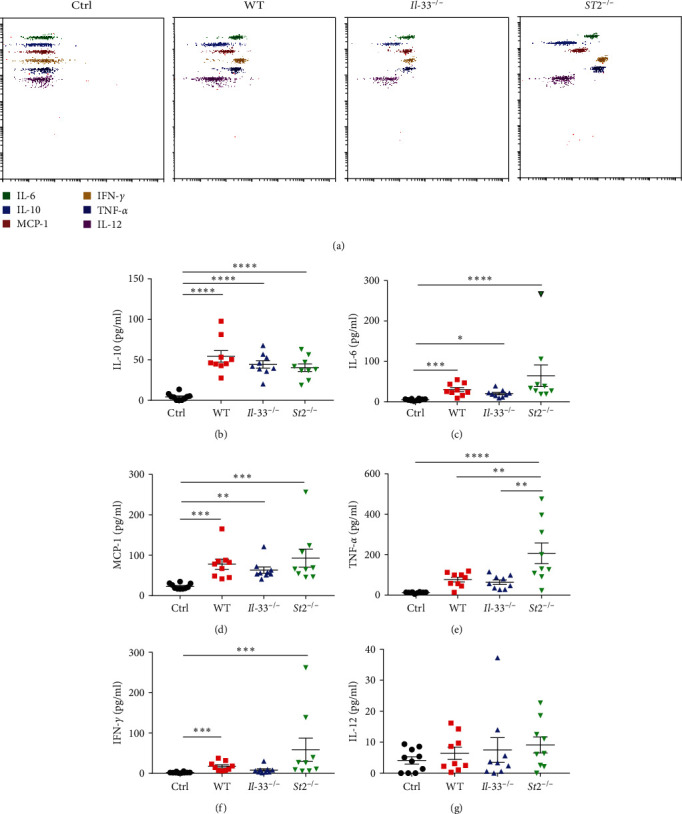
Cytokine levels in the four groups of mice 10 days after the model. (a) CBA kit detected the changes in cytokine levels in four groups. (b–g) The levels of IL-10, IL-6, MCP-1, TNF-*α*, IFN-*γ*, and IL-12 in the peripheral blood of mice in four groups.  ^*∗*^*P* < 0.05,  ^*∗∗*^*P* < 0.01,  ^*∗∗∗*^*P* < 0.001,  ^*∗∗∗∗*^*P* < 0.0001.

To further address the roles of IL-33 and ST2 in our MAS mice model, *IL-33*^*−/−*^ and *St2*^*−/−*^ mice were used to induce this model. All three groups of mice (WT, *IL-33*^*−/−*^ and *St2*^*−/−*^ mice) induced with CpG had a significant loss of body weight compared to the control mice treated with PBS, but there was no difference between the three groups after CpG induction (Figure 2(a)). Liver and spleen data demonstrated significant increases in the organ index for all three groups of MAS mice compared to PBS-treated controls (Figure 2(b)–2(d)). The erythrocyte counts, hemoglobin levels, and platelet counts of the three groups were significantly lower than those of the control group (Figure 2(e)–2(g)). However, there was no difference in white blood cell counts (Figure 2(h)). Further detection of serum liver enzyme levels showed that ALT and AST levels were all increased in the model mice compared with the control group (Figures 2(i) and 2(j)). The IL-18 levels were evaluated by ELISA. Plasma concentrations of IL-18 in the *St2*^*−/−*^ group were significantly higher than those in the control group, while IL-18 in the *IL-33*^*−/−*^ and WT groups only had an increasing trend (Figure 2(k)). To evaluate the cytokine levels in peripheral blood, the CBA kit was used to detect the elevation of cytokines in the three groups of model mice (Figure 3(a)). Further analysis showed that IL-10, IL-6, MCP-1, TNF-*α*, and IFN-*γ* in *St2*^*−/−*^ mice were significantly higher than those in the control group, and IL-12 also tended to increase. Notably, the TNF-*α* level was also elevated in *St2*^*−/−*^ mice compared with WT and *IL-33*^*−/−*^ mice (Figure 3(b)–3(g)).

### 3.4. Changes of Liver Inflammation in the IL-33^−/−^ and St2^−/−^ Mice

**Figure 4:**
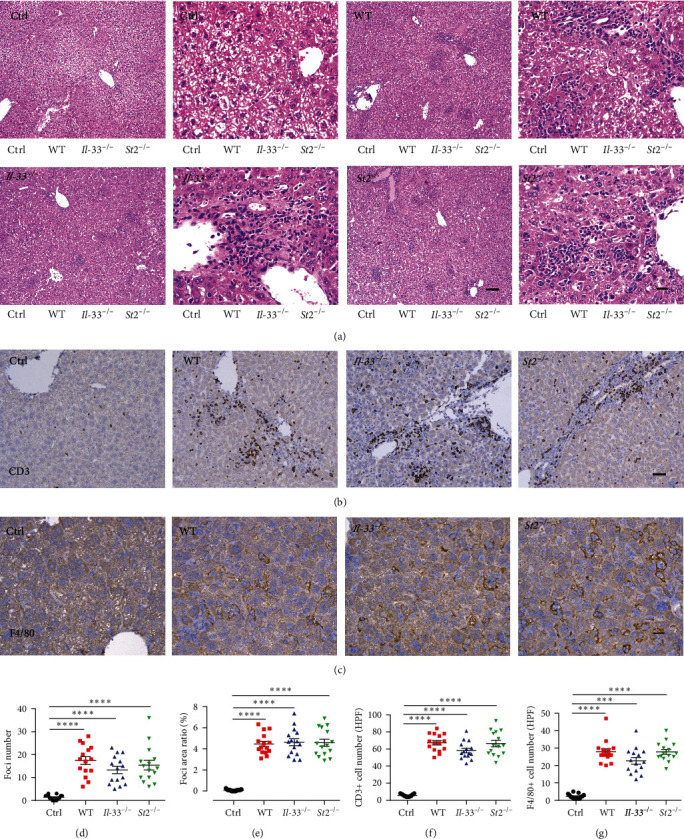
Liver inflammation levels and the number of CD3 and F4/80 in the four groups of mice 10 days after the model. (a) The liver H&E in four groups of mice (low magnification, bar = 200 *μ*m; high magnification, bar = 20 *μ*m). (b) Immunohistochemical staining of liver CD3 in four groups of mice (bar = 50 *μ*m). (c) Immunohistochemical staining of F4/80 in the liver of four groups of mice (bar = 20 *μ*m). (d and e) The foci number and foci area in the liver in four groups of mice. (f) CD3 positive cell counts in the liver at high magnification in four groups of mice. (g) F4/80 positive cell counts in the liver at high magnification in four groups of mice.  ^*∗∗∗*^*P* < 0.001,  ^*∗∗∗∗*^*P* < 0.0001.

Since the *IL-33*^*−/−*^ and *St2*^*−/−*^ mice failed to show significant phenotypical alterations compared with the WT mice that received CpG induction, the changes in liver inflammation in different groups of mice were then examined. First, the H&E staining was performed on the liver sections, and there was obvious inflammatory cell infiltration in the MAS mice. Statistical analysis showed that foci numbers and foci area in the MAS mice significantly increased compared with those in the control mice, but there were no significant differences among the three groups of MAS mice (Figures 4(a), 4(d), and 4(e)). Other inflammatory cells such as CD3 or F4/80 were also evaluated by immunohistochemistry. We found that CD3 was significantly increased in model mice, and the *IL-33*^*−/−*^ and *St2*^*−/−*^ mice did not interfere with CD3 infiltration in the liver (Figures 4(b) and 4(f)). Similar changes were also found in the F4/80 assessment (Figures 4(c) and 4(g)). These results suggest that whole-body knockout of *IL-33* or *St2* in mice has a limited effect on the infiltration of inflammatory cells in the liver in the CpG-induced MAS model.

### 3.5. Spleen Macrophages and Inflammatory Macrophage Levels

**Figure 5:**
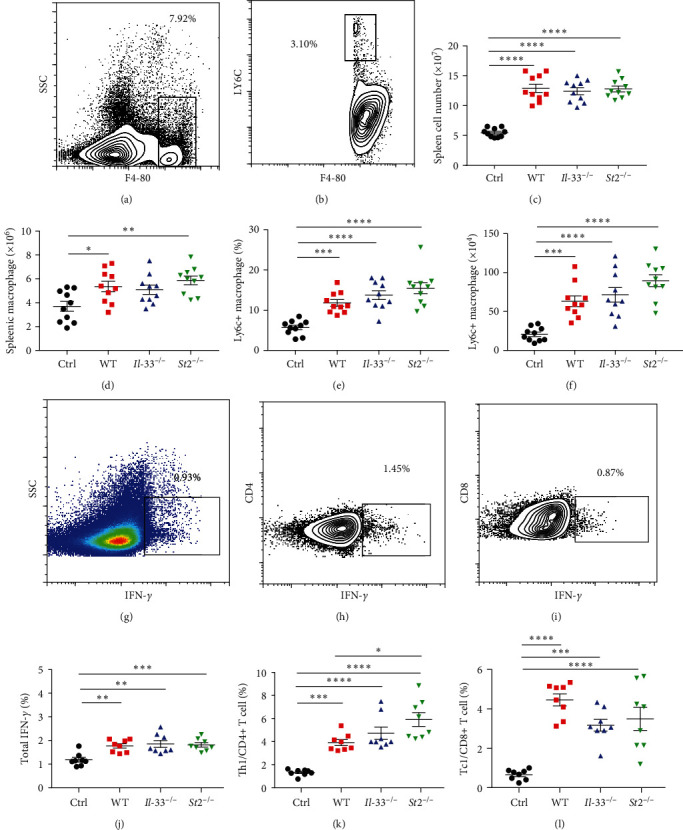
Ly6C+ macrophage counts and IFN-*γ* levels in the spleen of the four groups of mice 10 days after the model. (a) The levels of F4/80+ macrophages were analyzed by flow cytometry. (b) The proportion of Ly6C+ macrophages. (c and d) The total number of spleen cells and the number of macrophages in the four groups of mice. (e and f) The proportion and number of Ly6C+ macrophages in four groups of mice. (g–i) IFN-*γ* levels in the spleen, CD4+ T cells, and CD8+ T cells. (j–l) Statistical analysis of total IFN-*γ*, IFN-*γ*+ CD4+ T cells, and IFN-*γ*+ CD8+ T cells in four groups of mice.  ^*∗*^*P* < 0.05,  ^*∗∗*^*P* < 0.01,  ^*∗∗∗*^*P* < 0.001,  ^*∗∗∗∗*^*P* < 0.0001.

In addition to the liver, the inflammatory alterations in the spleen were subsequently tested. The macrophages were analyzed by F4/80 in the splenic single-cell suspension, and inflammatory macrophages were evaluated by Ly6c determination (Figures 5(a) and 5(b)). The results showed that the total number of splenic cells increased significantly in the model mice compared with the control group (Figure 5(c)). Further analysis showed that the total splenic macrophages of WT and *St2*^*−/−*^ mice increased significantly compared with the control group, while *IL-33*^*−/−*^ groups only showed an increasing trend (Figure 5(d)). Further analysis found that the proportion and number of inflammatory macrophages in model mice were significantly increased compared with the control mice (Figures 5(e) and 5(f)).

### 3.6. IFN-*γ*+ T Cells and Effector Memory T Cells in the Spleen

**Figure 6:**
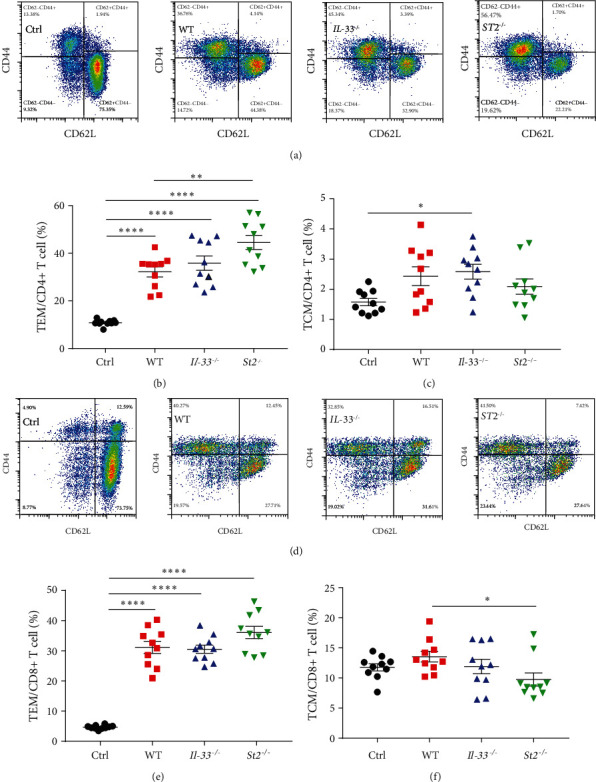
TEM and TCM levels in spleen cells of the four groups of mice 10 days after the model. (a) TCM and TEM in CD4+ T cells were analyzed by flow cytometry. (b and c) The proportions of TEM and TCM in CD4+ T cells. (d) The proportions of TEM and TCM in CD8+ T cells. (e and f) The proportions of TEM and TCM in CD8+ T cells.  ^*∗*^*P* < 0.05,  ^*∗∗*^*P* < 0.01,  ^*∗∗∗∗*^*P* < 0.0001.

T cell activation and their released IFN-*γ* have been demonstrated to play key roles in the pathogenesis of MAS. Therefore, the proportion of IFN-*γ*+ T cells in the spleen was analyzed. We found that *IL-33*^*−/−*^ and *St2*^*−/−*^ did not affect the ratio of total IFN-*γ*+ cells (Figures 5(g) and 5(j)). Further analysis of the proportions of CD4+ and CD8+ IFN-*γ*+ T cells showed that compared with WT mice, *St2*^*−/−*^ mice had a higher proportion of CD4+ IFN-*γ*+ T lymphocytes, and *IL-33*^*−/−*^ mice also tended to have increased frequency of CD4+ IFN-*γ*+ T lymphocytes (Figures 5(h) and 5(k)). The proportions of CD8+ IFN-*γ*+ T cells in *IL-33*^*−/−*^ and *St2*^*−/−*^ mice tended to decrease but without the difference (Figures 5(i) and 5(l)). We then assessed the proportions of effector (TEM, identified as CD44+CD62L−) and central memory T cells (TCM, identified as CD44+CD62L+) in CD4+ and CD8+ T cells. Compared with the control group, the ratio of TEM of CD4+ in model mice increased, but the ratio of TEM of *St2*^*−/−*^ mice was higher than that of WT mice (Figures 6(a) and 6(b)). Only *IL-33*^*−/−*^ mice had increased CD4+ TCM compared with the control group (Figure 6(c)). For CD8+ T cells, compared with the control group, the proportion of TEM in the three MAS groups was significantly increased, but TCM in WT mice was significantly higher than that in *St2*^*−/−*^ (Figure 6(d)–6(f)). These results indicate that IL-33 and ST2 affect IFN-*γ*+ T cell subtypes but not the total IFN-*γ*+ cells. In addition, in the model of *IL-33*^*−/−*^ or *St2*^*−/−*^ mice, the ratio of TEM and TCM is also very high, especially in *St2*^*−/−*^ mice.

## 4. Discussion

MAS is a potentially fatal disease caused by the overactivation of T lymphocytes and macrophages. It often leads to cytokine storms and a hyperinflammatory state. Clinically, MAS is more common in infections, malignant tumors, acquired immune disorders, and various autoimmune diseases (including juvenile idiopathic arthritis, systemic lupus, Kawasaki disease, and so forth). Currently, treatments for MAS are limited, and patients often have a high risk of death. The traditional therapies mainly focused on blocking T cell proliferation and activation with drugs such as dexamethasone or etoposide [9]. However, more new therapeutic approaches are already being developed. In particular, JAK inhibitors such as Ruxolitinib have shown extraordinary therapeutic potential in preclinical mouse models [26]. Nevertheless, a better understanding of the pathophysiology of the disease under different triggers is essential.

IL-33 is a chromatin-related nuclear cytokine, which can play a variety of extracellular biological effects in full-length or truncated active forms [24]. IL-33 is believed to be involved in cytokine storms induced by a variety of pathogens, including influenza virus [27], Group A Streptococcus (GAS) [28], LCMV virus [25, 29], COVID-19 [30], and so forth. However, the role of IL-33 in these diseases is inconsistent. For example, infection with the influenza virus can cause the epithelial cells to release IL-33 and promote the repair of lung tissues by inducing the production of AREG in ILC2 and Tregs; In the air pouch of GAS-infected mice, the IL-33/ST2 axis also has a protective effect by enhancing the migration and activity of neutrophils; In LCMV-infected *Prf1*^*−/−*^ or *IFNγ*^*−/−*^*Prf1*^*−/−*^ mice, the IL-33/ST2 axis were proved to ameliorate the manifestations of HLH/MAS; In COVID-19 infection, IL-33 is not only associated with lung injury but also stimulated T cell activity and antibody production, which exert some benefits. These results suggest that IL-33 is actively involved in cytokine storm-related diseases. IL-33 is pleiotropic, and its function depends on the characteristics of the disease's immune microenvironment.

MAS can be induced by a variety of factors and different triggers, the immune microenvironment may be different. Although studies support the pathogenic role of IL-33/ST2 in LCMV-infected *Prf1*^*−/−*^ mice, the specific role of the IL-33/ST2 axis is not clear in the CpG-induced MAS model. Considering the pleiotropic and complex nature of IL-33 and some patients with MAS induced by pathogen infection in clinical practice, we detected the expression of IL-33 and ST2 in CpG-induced MAS mice and further verified it in knockout mice.

Our results showed that after repeated injection of CpG, IL-33 levels in both the liver and spleen decreased, while ST2 tended to increase. Therefore, IL-33 and ST2 may be actively involved in the development of the disease. However, *IL-33*^*−/−*^ or *St2*^*−/−*^*mice* did not improve hypercytokinemia, hepatosplenomegaly, or cytopenia in the CpG-induced MAS model. *St2*^*−/−*^ mice even had an aggravation trend. These results are essential and significant. It shows that the effects of IL-33 and ST2 are inconsistent in different models with different causes. IL-33 and ST2 may be more important in the disease condition which is more IFN-*γ* dependent. Therefore, treatment with ST2-neutralizing antibodies can be beneficial in primary HLH, but not in the CpG-induced model where the up-regulation of IFN-*γ* was minimal. Although IL-33 and ST2 may also be involved in disease, they are not the determinants of disease occurrence and progression. These results also suggest that targeting IL-33 and ST2 should be cautious for CpG-induced MAS.

Our results have implications and references for the precise treatment of disease. However, more information about the pathophysiological mechanism of MAS syndrome and the cytokine levels involved needs to be further explored. It is hoped that there will be more evidence of MAS-related treatments in the future.

## Data Availability

The data supporting the conclusions of this article will be made available by the authors, without undue reservation.

## Supplementary Materials

Supplementary Materials Figure S1: hepatosplenomegaly and cytokine storm in mice 10 days after the model.
