# Supplementary material for: The Role of the IL-33/ST2 Axis in CpG-Induced Macrophage Activation Syndrome
Source: J Immunol Res. 2023 Oct 5;2023:2689360. doi: 10.1155/2023/2689360 (PMC10569892; doi:10.1155/2023/2689360)
Supplement: Supplementary Materials — Figure S1: hepatosplenomegaly and cytokine storm in mice 10 days after the model. [file 2689360.f1.docx]

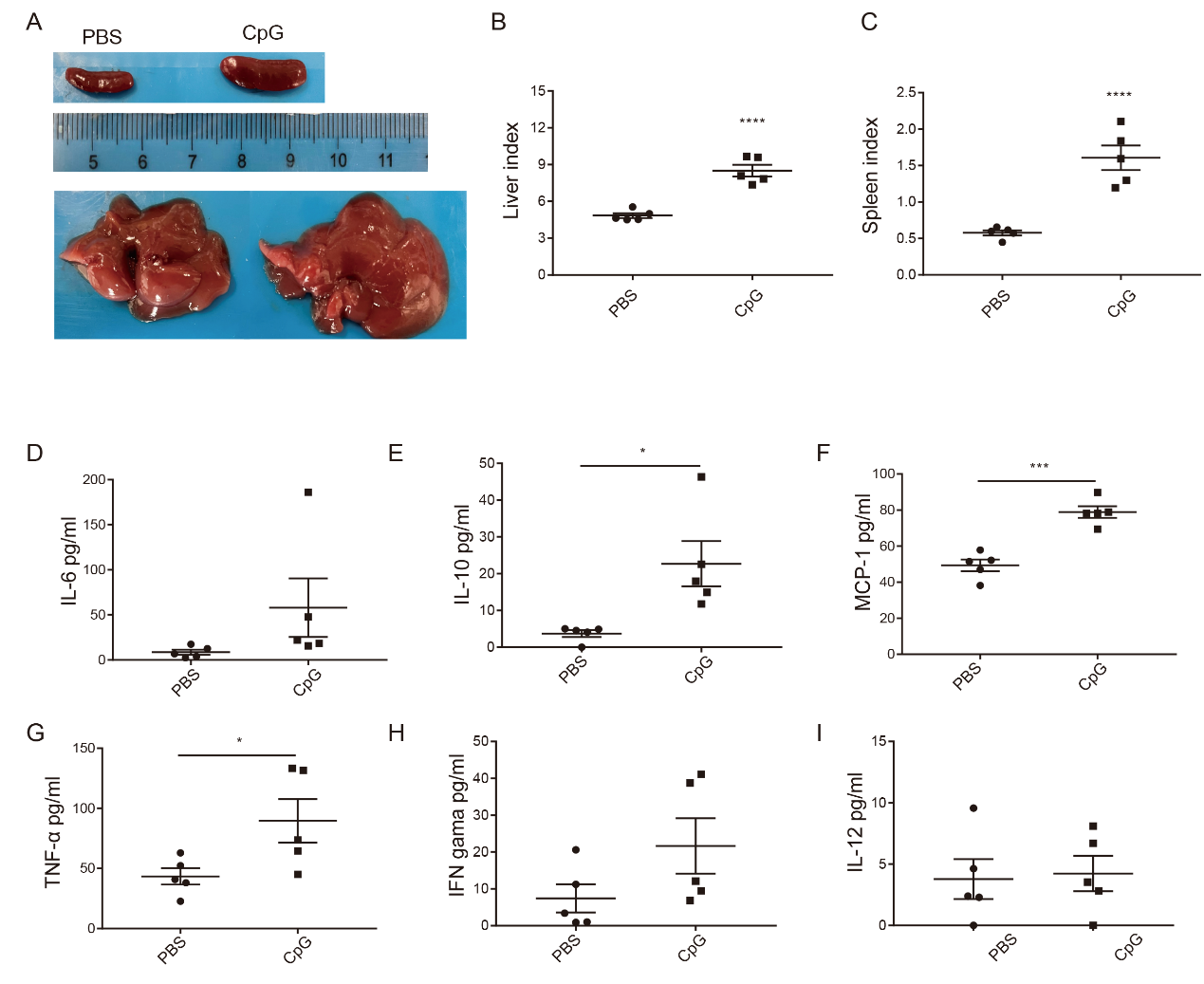


Supplementary Figure S1: Hepatosplenomegaly and cytokine storm in mice ten days after the model. (A) Images of the liver and spleen of control mice and mice repeatedly stimulated with CpG. (B) The liver index increased significantly in mice that received repeated CpG stimulation. (C) The spleen index was significantly increased in mice with repeated CpG stimulation. (D-I) The levels of IL-6, IL-10, MCP-1, TNF-α, IFN-γ, and IL-12 cytokines in the peripheral blood of control mice and mice repeatedly stimulated with CpG. (**P*<0.05， ****P*<0.001，**** *P*<0.0001)
